# A glutamate receptor-like gene is involved in ABA-mediated growth control in *Physcomitrium* (*Physcomitrella) patens*

**DOI:** 10.1080/15592324.2022.2145057

**Published:** 2022-11-20

**Authors:** Ya Wang, Dongsheng Yu, Hongmiao Zhao, Lanlan Jiang, Lei Gao, Yanan Song, Zebin Liu, Fang bao, Congcong Hou, Yikun He, Chuanli Ju, Legong Li, Dongdong Kong

**Affiliations:** Key Laboratory of Plant Gene Resources and Biotechnology for Carbon Reduction and Environmental Improvement, Beijing Municipal Government, and College of Life Sciences, Capital Normal University, Beijing, China

**Keywords:** Glutamate receptor homolog, abscisic acid, growth control, moss, gene expression

## Abstract

Plant glutamate receptor homologs (GLRs), which function as key calcium channels, play pivotal roles in various developmental processes as well as stress responses. The moss *Physcomitrium patens*, a representative of the earliest land plant lineage, possess multiple pathways of hormone signaling for coordinating growth and adaptation responses. However, it is not clear whether GLRs are connected to hormone-mediated growth control in the moss. In this study, we report that one of the two GLRs in *P. patens*, PpGLR1, involves in abscisic acid (ABA)-mediated growth regulation. ABA represses the growth of wild-type moss, and intriguingly, the *PpGLR1* transcript levels are significantly increased in response to ABA treatment, based on both gene expression and the *PpGLR1pro::GUS* reporter results. Furthermore, the growth of *Ppglr1* knockout moss mutants is hypersensitive to ABA treatment. These results suggest that PpGLR1 plays a critical role in ABA-mediated growth regulation, which provide useful information for our further investigation of the regulatory mechanism between Ca^2+^ signal and ABA in moss growth control.

## Introduction

As one of the most primitive and simple non-vascular plants, bryophytes occupy a unique evolutionary position as the intermediate between green algae and vascular plants. The bryophyte *P. patens* belongs to the Funariaceae and has been used as a typical representative of early terrestrial plants in the past 50 years.^1–3^ As the first genome-sequenced non-vascular plant, the availability of the whole genome sequence,^4^ together with efficient gene manipulating techniques, makes *P. patens* a suitable species for addressing key questions in relation to plant development and stress responses.

Being the earlier land plants in terrestrial conditions, *P. patens* has to maintain normal growth and cope with complicated environmental changes. Multiple signaling pathways are required to ensure normal plant developmental and adaptation processes,^1–3^ among which calcium acts as a versatile signaling molecule due to its instant response to endogenous and external stimuli.^5,6^ Under the stimuli, calcium channels distributed on the cytoplasmic membrane or endomembrane system can be transiently activated, resulting in a fluctuation of cytosolic-free calcium ions, known as Ca^2+^ signature, and then a further plant growth regulation process.^7^

Based on sequence homology search of animal calcium channels in the plant genomes, putative candidates of plant calcium channels were identified, mainly including cyclic nucleotide-gated ion channels (CNGCs), glutamate receptor-like channels (GLRs), and calcium permeable stress-gated cation channels/reduced hyperosmolality-induced [Ca^2+^]_i_ increase channels (CSCs/OSCAs) and so on.^8–11^ Subsequent studies have shown that these genes function in a variety of Ca^2+^-dependent physiological or pathological processes.^6,7,12,13^

Since the discovery of putative *GLRs* in the model plant *Arabidopsis* in 1998,^8^ studies have demonstrated that *GLRs* involve in various physiological processes in different plant species.^7,14^ With the advances of studies in molecular genetics, cell biology, and plant physiology, the functions and molecular regulatory mechanisms of plant *GLRs* have attracted extensive attention, with their roles covering normal developmental programs, such as C/N metabolism, root growth and development, seed germination, stomatal movement, and pollen tube morphogenesis and fertilization,^14–21^ as well as stress responses.^22–26^ The report that two *GLRs* in the moss *P. patens, PpGLR1* and *PpGLR2*, function in sperm tropism and reproduction greatly expands our understanding about the roles of GLRs in the early land plant species.^14^ However, whether *PpGLRs* are implicated in the other process is largely unknown. In this study, we report that PpGLR1 is connected to the growth regulation performed by plant hormone abscisic acid (ABA).

## Materials and methods

### Plant materials and growth conditions

In this study, the *P. patens* (Hedwig) ecotype ‘Gransden 2004’ is used as the wild-type (WT) moss, and the *P. patens glr1* mutant lines were generated by CRISPR/Cas9-mediated gene editing approach in this study. The tissue of WT *P. patens* and the *Ppglr1* mutant was cultured on BCDA (for protonemata) or BCD (for gametophytes) medium supplemented with 0.5% (w/v) glucose and grown at 25°C under 120 μmol m^−2^ s^−1^ light with a long-day photoperiod (16-h light/8-h dark). For growth phenotype analysis, the tips of WT gametophytes with uniform size were cultured on BCD medium supplemented with indicated concentrations of the hormone for 7 weeks. The same stage *P. patens* samples were harvested for reverse transcription-PCR (RT-PCR) analysis. For the expression analysis of *PpGLR1* and *PpGLR2* in young tissue, gametophore tissues of WT were homogenized gently, and the obtained protonemata was cultured on BCDA medium for two weeks with a second time homogenization in the middle. The protonemata was then transferred to cellophane-overlayed BCD medium for gametophore growth and collected at indicated time points for the expression analysis.

### Gene expression analysis

Total RNA of the *P. patens* tissue was extracted using Spectrum Plant Total RNA Kit (Sigma) and reverse transcribed using iScript cDNA Synthesis Kit (Bio-Rad) after the treatment with DNase I (Ambion). For semi-quantitative RT-PCR analysis of *PpGLR1* and *PpGLR2* expression in gametophytes, PCR amplifications were performed with 31 cycles for *PpGLR1* and 28 cycles for *PpGLR2* and *Tubulin* respectively, using the following gene-specific primers: *PpGLR1*, 5’-GACCCCAAATTTTCGCAGGCACTC-3’ and 5’-GAGCTGCAGCAGGTAGTCCCGCAC-3’; *PpGLR2*, 5’-GGACAAAGACTCAGATTTTCGGC-3’ and 5’-GAAGGATCCCGTTTGGTAGCCGATAG-3’; and *Tubulin*, 5’-GAGTTCACGGAAGCGGAGAG-3’ and 5’-TCCTCCAGATCCTCCTCATA-3’. qRT-PCR was conducted with SYBR Green PCR Master Mix (Bio-Rad) using the CFX96 Real-Time PCR System (Bio-Rad), and relative expression level was normalized to that of *Tubulin*. Primers used for *PpGLR1* and tubulin genes are as follows: *PpGLR1*, 5’-TTCTTCACGGGTCTAGTAGTGTGG-3’ and 5’-GAGCTGCAGCAGGTAGTCCCGCAC-3’; and *tubulin*, 5’-GAGTTCACGGAAGCGGAGAG-3’ and 5’-TCCTCCAGATCCTCCTCATA-3’. For the expression analysis, three biological replicates were conducted, and the data presented are from one experimental repetition with three technical repeats.

### *Generation of the* Ppglr1 *mutant and* P. patens *transgenic plants*

The *Ppglr1* mutant generation and related protoplast preparation and stable transformation were performed using standard PEG-mediated approach. To generate the *Ppglr1* mutant lines, we subjected the first exon of *PpGLR1* to a search of CRISPR RNA (ATTGTCGCTGGAGGGCTAAC) with the webtool CRISPOR V1 to the *P. patens* genome Phytozome V9 http://crispor.tefor.net/crispor.py). The sgRNA constructed a 20 bp CRISPR RNA of *PpGLR1* fused with 83 bp of the *S. pyogenes* tracrRNA scaffold under the U6 promoter control as described previously.^27^ Transformants of the *Ppglr1* mutant candidates were selected on BCD medium supplemented with 50 mg/L G418, and confirmed by sequencing of the target *PpGLR1* fragment using primers 5’-ACTGAATCGTGGGGTTCGAC-3’ and 5’-GCCCAGATGTAGCACCAACT-3’. To conduct the *PpGLR1pro::GUS* reporter assay, a 2-kb fragment of the *PpGLR1* promoter was amplified from WT genomic DNA with specific primers *PpGLR1pro-F* 5’-CGGTACCAGGGTGCTAAAGATCACTTTATTTGG-3’ and *PpGLR1pro-R* 5’-GGGCGCGCCACTTTCCTTGGTACTGTTTACTTCTAAACC-3’, and cloned into *pTFH15.3-GUS* vector with restriction enzyme sites of *KpnI* and *AscI* then performed protoplast transformation. To select transgenic plants of *PpGLR1pro::GUS*, transformed protoplasts were selected on BCD medium supplemented with 50 mg/L G418. After a period of 6-week regeneration, the candidate lines were examined for GUS signal.

### Histochemical GUS activity analysis

GUS activity was examined according to the procedure described.^28^ Briefly, the *PpGLR1pro::GUS* transgenic lines were incubated in GUS staining buffer at 37°C in the dark overnight and cleaned with 70% (v/v) ethanol to remove chlorophyll. Samples from control or hormone-treated medium were stained and decolorized at the same time for result comparison. Photographs were taken using a Zeiss Axio Zoom V16 equipped with a Zeiss Axio CamICc digital camera.

### Statistical analysis

Student’s t-test (one-tailed distribution) was used to determine the significance of the data.

## Results

**Figure 1.**
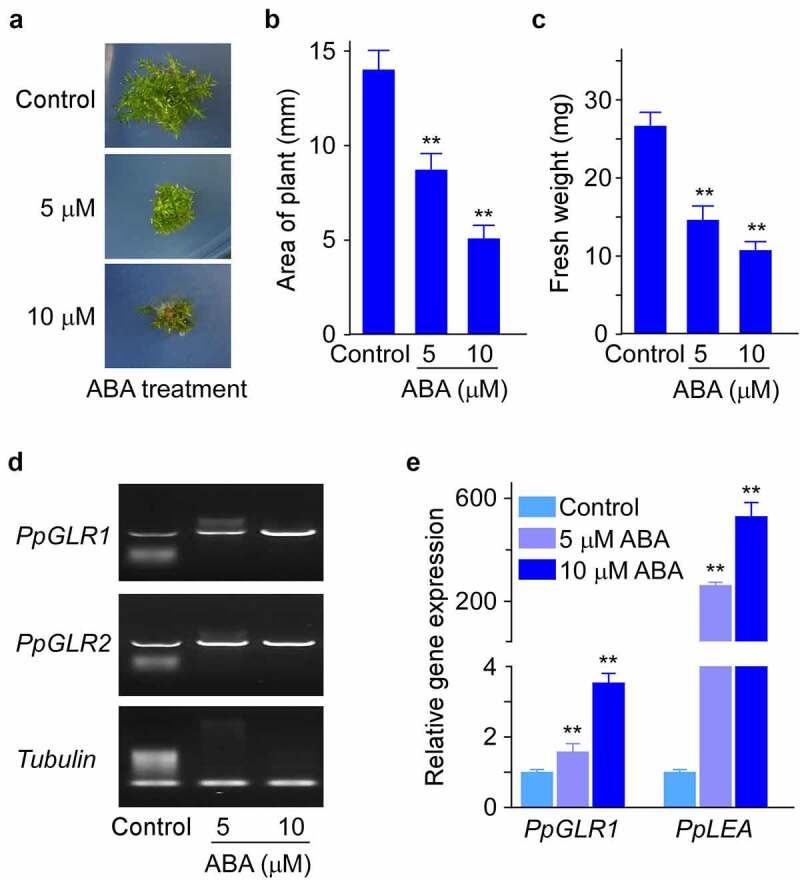
The expression analysis of two *PpGLRs* under different concentrations of ABA in *P. patens*. (a) Phenotypes of wild-type (WT) *P. patens* cultured on BCD medium supplemented with indicated concentrations of ABA. Three biological replicates were done, and representative pictures are shown. (b and c) Statistical analyses of the **area** and fresh weight of the gametophyte under indicated amounts of hormone. Three biological replicates were done, and data shown are mean of thirty samples from one replicate and error bars indicate SD. (d) semi-quantitative RT-PCR analysis of the expression of *PpGLR1* and *PpGLR2* under indicated concentration of hormone treatment. *Tubulin* was used as a loading control. (e) qRT-PCR analysis of *PpGLR1* and *PpLEA* expression under different concentrations of ABA. Three biological replicates were done and similar gene expression trends are shown. Data from three technical repeats are shown, and error bars represent SD. In B, C, and E, ** indicates significant differences between control and hormone-treated sample (*P* < .01).

To examine the effects of plant hormone ABA on the growth of *P. patens*, we cultured WT *P. patens* under indicated amounts of ABA and analyzed the growth phenotypes. Compared with control, ABA inhibited *P. patens* growth in a dose-dependent manner, as shown by the significantly decreased plant size (as shown by the area of gametophyte) and fresh weight (Figure 1a–c). Compared with ABA treatments, GA treatment caused no visible differences in plant size (Supplementary Figure S1). We next examined whether the two *PpGLRs* were connected to the hormone-modulated growth control in *P. patens*, and we detected the expression levels of *PpGLR1* and *PpGLR2* in the above hormone-treated samples by semi-quantitative RT-PCR analysis. The results showed that the expression levels of *PpGLR1* were obviously increased by ABA treatments (Figure 1d). In contrast, the expression levels of *PpGLR2* showed no clear changes in response to either of the hormone treatment (Figure 1d and Supplementary Figure S2), which may imply its more specific roles in the sexual reproduction as reported previously.^14^ Consistent with the observed ABA-induced *PpGLR1* transcript increase pattern (Figure 1d), qRT-PCR results showed a dose-dependent increase of ABA effects on *PpGLR1* expression levels (Figure 1e). In line with the above results, the transcript amounts of *LATE EMBRYOGENESIS ABUNDANT* (*PpLEA*), a gene known to be involved in ABA response, were greatly increased in the ABA treated samples (Figure 1e), supporting the reliability of our experimental conditions.

**Figure 2.**
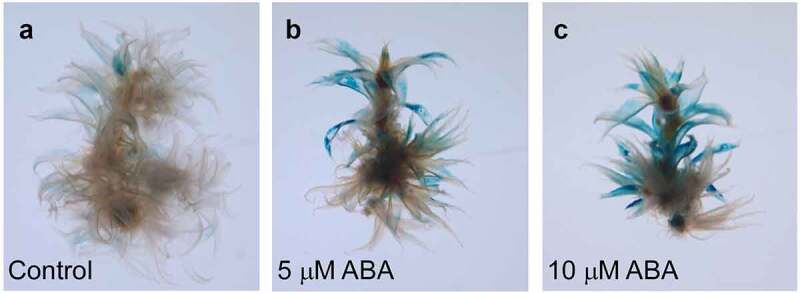
*PpGLR1pro::GUS* reporter analysis of the *PpGLR1* expression under ABA treatments. Gametophytes of the *PpGLR1pro::GUS* transgenic lines were cultured under the control and indicated concentration of the hormone for fifteen days and collected for the analysis. Representative pictures are shown.

We further characterized the expression sites of *PpGLR1* using the *PpGLR1pro::GUS* transgenic plants. We cloned a 2-kb promoter sequence of *PpGLR1*, fused it with the *GUS* reporter gene, and then generated the *PpGLR1pro::GUS* transgenic plants in *P. patens*. Histochemical GUS activity analysis showed that *PpGLR1* transcript was mainly detected in the phyllids of the gametophytes under normal culture conditions (Figure 2a). In response to 5 or 10 µM ABA treatment, *PpGLR1* transcript was highly up-regulated, in the same expression sites as the control (Figure 2b,c). Compared with the control, no obvious changes of *PpGLR1pro::GUS* activity were observed under GA treatments (Supplementary Figure S3). Thus, both gene expression and the *PpGLR1pro::GUS* reporter analysis results demonstrate that the transcript amounts of *PpGLR1* increase obviously in response to ABA. The expression level of *PpGLR1* increases while the growth of *P. patent* is repressed by ABA treatments, suggesting that *PpGLR1* plays a role in ABA-regulated growth control in *P. patens*.

Furthermore, to know more about the physiological roles of *PpGLR1* in *P. patens*, we examined the transcript levels of *PpGLR1* in protonema. WT *P. patens* tissue was collected respectively at four time points (0 d, 3 d, 7 d, and 10 d) after transfer to the new medium, and the expression level of *PpGLR1* was analyzed by semi-quantitative RT-PCR. The results showed that *PpGLR1* exhibited a first increase and then a decrease trend during the time points detected (Supplementary Figure S4), which may suggest a regulatory role of *PpGLR1* in protonema growth regulation of *P. patens*.

**Figure 3.**
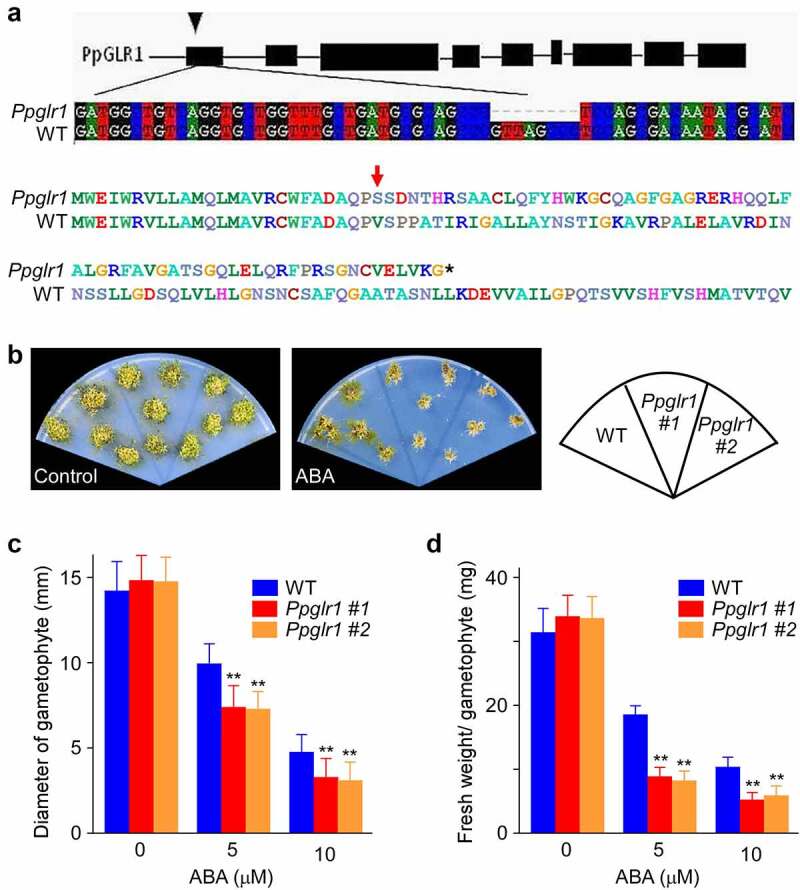
The growth of *Ppglr1* mutant was hypersensitive to ABA. (a) The gene structure of *PpGLR1* (top) and the sequencing results of the *PpGLR1* gene in the *Ppglr1* mutant showing an 8 bp deletion which would lead to the premature termination of the PpGLR1 protein translation (bottom). (b) Phenotypes of WT and the *Ppglr1* mutant lines under the control and ABA treatment. Gametophytes of WT and *Ppglr1* mutants were cultured on BCD medium supplemented with 0 or 5 µM ABA for 7 weeks. Three independent experiments were performed, and representative images are shown. (c and d) Statistical analysis of the diameter and fresh weight of the gametophyte from WT and *Ppglr1* mutants under indicated amounts of ABA. Data from thirty samples were analyzed under each treatment, and error bars indicate SD. ** indicates significant differences between WT and *Ppglr1* mutant under the same condition (*P* < .01).

To assess the role of *PpGLR1* in ABA-mediated *P. patens* growth control, we generated *Ppglr1* knockout mutants using CRISPR/Cas9 method and tested their phenotypes under ABA treatment. The *Ppglr1* mutants contain an 8 bp nucleotide deletion (Figure 3a), and this would lead to the premature termination of the PpGLR1 protein translation. WT and *Ppglr1* mutants were grown on BCD medium supplemented with 0 or 5 µM ABA for 7 weeks for phenotype analysis. Under normal conditions, the *Ppglr1* mutants were similar to WT in terms of plant size and fresh weight (Figure 3b–d). In contrast, under ABA treatment, the mutants were smaller and browser than wild type (Figure 3b), with the plant size and fresh weight being reduced to 75% and 45% of the WT, respectively (Figure 3c,d). These results indicate a correlation between the *PpGLR1* expression level and ABA sensitivity in terms of *P. patent* growth phenotype.

## Discussion

Calcium channel-mediated calcium signal plays pivotal roles in plant growth and development.^5–7^ Increasing evidence has demonstrated that GLR channels function via generating and transducing specific cytosolic calcium signals in different plant species.^28,29^ A recent study in *P. patens* showed that two *PpGLRs* served as key regulators of Ca^2+^ signal in sperm chemotaxis and transcriptional regulation,^14^ which extends our knowledge about *GLR* function to the extremophile land plant. In the current work, through a combination of CRISPR/Cas9-mediated gene editing approach, phenotypic observation, and gene expression analysis, we demonstrate that one of the two *PpGLRs, PpGLR1*, plays an important role in phytohormone ABA-modulated growth control in the moss.

Compared with the large GLR gene family with big members in seed plants, the genome of the early land plant *P. patens* contains only two GLRs, *PpGLR1* and *PpGLR2*.^29,30^ These two PpGLRs therefore may execute relatively conserved function compared to the counterparts in seed plants that have various specialized function. We show that the transcript of *PpGLR1* but not *PpGLR2* responds to and accumulates under ABA treatment (Figures 1d–e and 2 and Supplementary Figure S2). Our results, together with the previous finding that *PpGLR1* and *PpGLR2* showed different spatiotemporal expression pattern,^14^ indicate that the two PpGLRs have distinct function. The hypersensitive growth phenotype of the *Ppglr1* mutant under ABA treatment (Figure 3) suggests that *PpGLR1* is a regulator in ABA-mediated developmental process in the moss *P. patens*. Interestingly, studies have demonstrated that repressing the function of several *Arabidopsis GLRs* impaired cytosolic Ca^2+^ concentration and ABA sensitivity,^20,31,32^ and these *Arabidopsis* GLRs all belong to subfamily III, one of the three subfamilies divided in *Arabidopsis*. From a phylogenetic point of view, two *PpGLRs* are close to the most ancient *GLR* subfamily III (Clade III) from land plants, particularly in the conserved amino-terminal domain (ATD).^29,30,33^ Recently, several studies have reported that altering the expression level of GLR changed ABA sensitivity in Arabidopsis.^20,31,32^ These studies are in line with the results shown in *P. patents* (Figure 3, b-d), which together suggests that the regulatory connection between GLR-involved Ca^2+^ signals and phytohormone ABA-mediated growth exists in moss, the early land plants, and is conserved between *P. patens* and *Arabidopsis*. Moreover, our current research might also have indicated an important evolutionary conservation of GLR functional role in plants.

In this study, we show that the *Ppglr1* knockout moss mutant is hypersensitive to ABA in growth (Figure 3b–d), and consistent with our results, a recent report showed that overexpression of *Arabidopsis GLR3.7* alleviated ABA sensitivity in germination.^32^ However, our observation that the transcript of *PpGLR1* increased under ABA treatment (Figure 1) is inconsistent with their report showing that ABA treatment suppressed the expression of *GLR3.7*.^32^ This divergence could be attributable to differences in the treatment conditions and the GLR member studied. In addition, considering the universal roles of ABA signaling in a variety of stress responses, such as salinity, drought, temperature, and so on, and the cross regulation of plant GLRs in these adversity physiology against environmental menace, we speculate that *PpGLRs*, especially *PpGLR1*, may undertake more potential functions responding to adverse environmental stimuli. More related studies in the future would help us better understand the regulatory mechanism between GLR and ABA-mediated physiological process.

## Supplementary Material

Supplemental Material
