# Supplementary figures and images for: A glutamate receptor-like gene is involved in ABA-mediated growth control in Physcomitrium (Physcomitrella) patens
Source: Plant Signal Behav. 2022 Nov 20;17(1):2145057. doi: 10.1080/15592324.2022.2145057 (PMC9677993; doi:10.1080/15592324.2022.2145057)

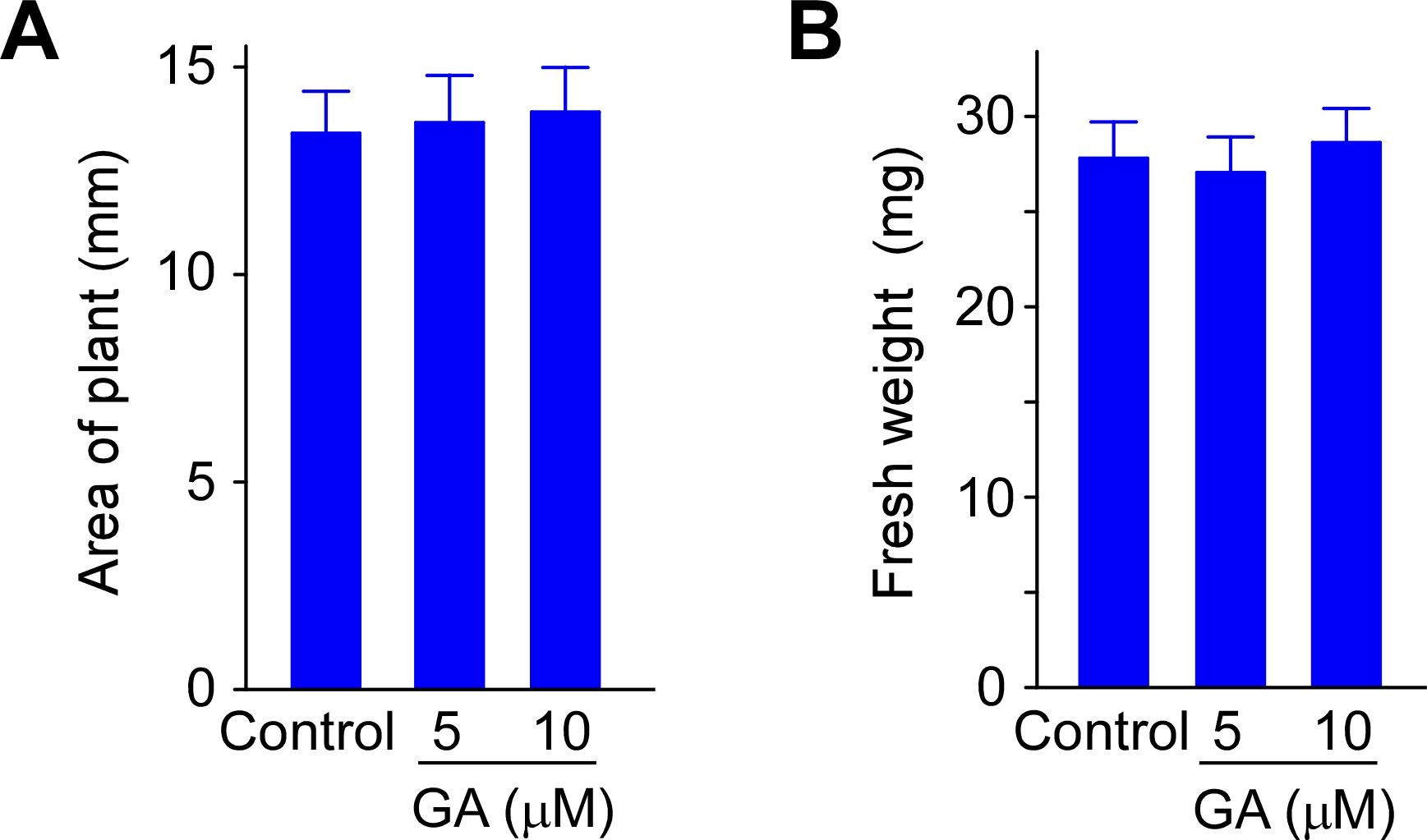

Supplement: Supplemental Material [file KPSB_A_2145057_SM7788.zip › Wang Supplementary Figure S1.jpg]

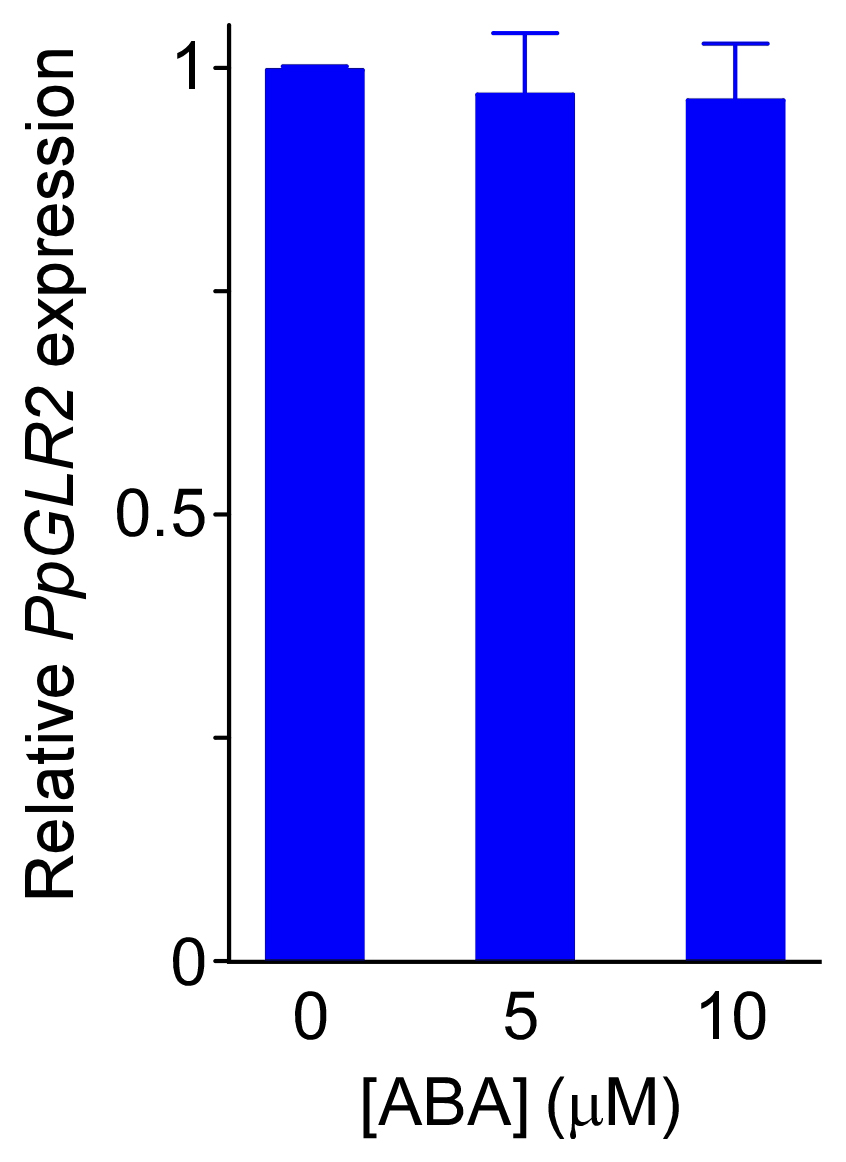

Supplement: Supplemental Material [file KPSB_A_2145057_SM7788.zip › Wang Supplementary Figure S2.jpg]

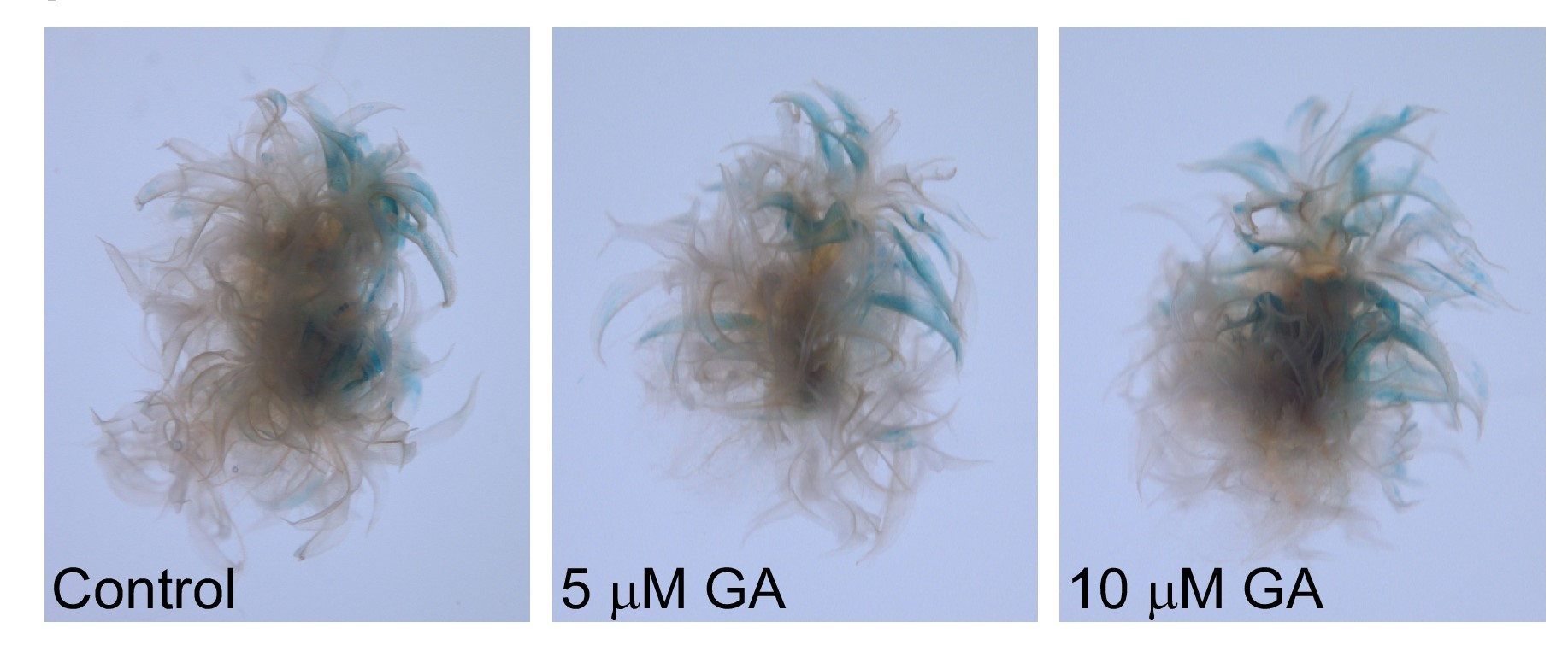

Supplement: Supplemental Material [file KPSB_A_2145057_SM7788.zip › Wang Supplementary Figure S3.jpg]

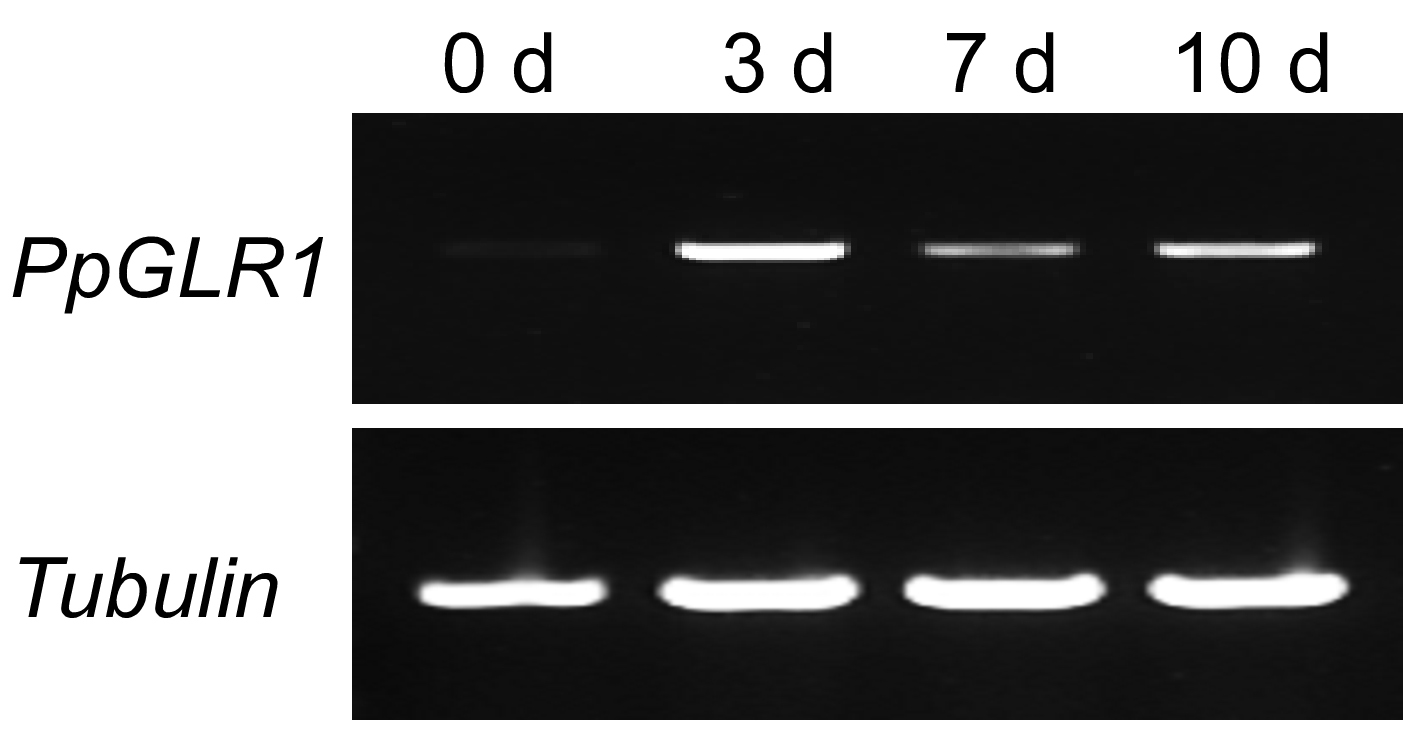

Supplement: Supplemental Material [file KPSB_A_2145057_SM7788.zip › Wang Supplementary Figure S4.jpg]
